# Systemic and organ-specific autoantibodies and worse outcomes in autoimmune encephalitis: a multicenter cohort study

**DOI:** 10.3389/fimmu.2026.1759213

**Published:** 2026-01-21

**Authors:** Xin Zhao, Ningning Wang, Tian Yang, Wenna Chen, Yajing Cheng, Teng Yao, Jing Wang, Run Song, Ganqin Du, Yaopeng Li

**Affiliations:** 1 Department of Neurology, The First Affiliated Hospital, and College of Clinical Medicine of Henan University of Science and Technology, Luoyang, China; 2 Department of Neurology, Tianjin Medical University General Hospital, Tianjin, China; 3 China National Clinical Research Center for Neurological Diseases, Beijing Tiantan Hospital, Capital Medical University, Beijing, China; 4 Department of Neurology, Affiliated Gaozhou People’s Hospital, Guangdong Medical University, Gaozhou, China

**Keywords:** autoimmune encephalitis, clinical features, outcomes, relapse, systemic and organ-specific autoantibodies

## Abstract

**Objective:**

Autoimmune encephalitis (AE) is a group of neuroinflammatory disorders with variable clinical outcomes. Despite the identification of specific neuronal autoantibodies, their clinical presentation and outcomes were significantly variable in clinical practice, suggesting the influence of additional factors beyond the primary autoantibody. Systemic and Organ-Specific autoantibodies (SAOS-Ab) are frequently detected in AE patients, but their clinical role remains incompletely characterized. We hypothesized that the coexistence of SAOS-Ab in AE was associated with a more severe disease course and higher relapse risk.

**Methods:**

In this multicenter, retrospective cohort study, we analyzed 218 AE patients from four Chinese tertiary centers (January 2018-October 2022). AE patients were stratified into SAOS-Ab-positive and SAOS-Ab-negative groups based on serological testing. We compared demographic, clinical, and immunological features. The primary outcomes were disease severity at onset (measured by clinical assessment scale in autoimmune encephalitis [CASE] scores), short-term functional outcome (measured by modified Rankin scale [mRS] scores), and 24-month relapse risk. Multivariable logistic and Cox regression analyses were employed to identify independent predictors of poor outcome and relapse.

**Results:**

A high prevalence (57.3%, 125/218) of SAOS-Ab was observed. The SAOS-Ab-positive group had a higher female proportion (55.2% vs 35.5%, *p* = 0.004), and elevated serum B cell proportions (*p* = 0.0027), IgG (*p* < 0.0001) and IgM (*p* = 0.0334). Clinically, these patients had higher initial CASE scores (*p* < 0.0001), greater ICU admission rates (21.6% vs 7.5%, *p* = 0.005), and more frequent poor functional outcomes at discharge (mRS ≥ 3; 47.2% vs 26.9%, *p* = 0.002). The 24-month relapse rate was significantly higher in the SAOS-Ab-positive group (29.8% vs 13.2%, *p* = 0.0043). After adjustment, SAOS-Ab positivity remained an independent risk factor for relapse (adjusted HR 2.270, 95% CI 1.174-4.387, *p* = 0.015).

**Conclusion:**

SAOS-Ab is a prevalent and clinically relevant biomarker in AE, associated with distinct immunologic profiles, more severe disease, and significantly increased relapse risk. These findings support the integration of SAOS-Ab testing into AE management to facilitate early risk stratification and more frequent follow-up.

## Introduction

Autoimmune encephalitis (AE) refers to a group of inflammatory neurological disorders characterized by the presence of autoantibodies targeting neuronal intracellular or cell-surface antigens, which are directed against antigens within the central nervous system (CNS). Key clinical manifestations include cognitive dysfunction, movement disorders, seizures, psychiatric symptoms, and decreased consciousness, with behavioral abnormalities and seizures being the most prevalent in both pediatric and adult cohorts (1, 2). The establishment of diagnostic criteria and the discovery of specific antibodies have enabled earlier diagnosis and immunotherapeutic intervention, significantly improving patient outcomes (3, 4). Despite these advancements, a substantial proportion of patients experience severe illness, incomplete recovery, or relapse (5). This clinical heterogeneity is observed not only across different antibody subtypes but also among individuals with identical neuronal autoantibodies, suggesting that factors beyond the primary antibody impact disease course (6).

AE frequently occurs alongside systemic autoimmune disorders or presents with serological evidence of autoimmunity in the absence of clinical disease (7, 8). These serological antibodies, collectively termed Systemic and Organ-Specific Autoantibodies (SAOS-Ab), include antinuclear antibodies (ANA), antibodies against extractable nuclear antigens (ENA), thyroid-specific antibodies, and others (9, 10). Previous studies have demonstrated the associations between ANAs and inflammatory neurological disorders, such as neuromyelitis optica spectrum disorder (NMOSD) and multiple sclerosis (MS) (11, 12). Recent studies have documented associations between ANA and anti-N-methyl-D-aspartate receptor (anti-NMDAR) encephalitis, linking their presence to greater disease severity and unfavorable outcomes (13, 14). However, those studies had small sample sizes, and the results of these studies were limited to ANA alone. Yet, the prevalence, clinical relevance, and prognostic implications of SAOS-Ab in AE remain inadequately characterized. We hypothesize that SAOS-Ab seropositivity signifies a state of generalized immune dysregulation in a subset of AE patients. This systemic autoimmunity may lead to a more severe initial inflammatory response within the CNS, contribute to treatment refractoriness, or increase the propensity for relapse. The detection of SAOS-Ab at diagnosis could serve as a valuable biomarker for risk stratification.

This study aims to comprehensively delineate the clinical and paraclinical characteristics of AE patients with coexisting SAOS-Ab seropositivity and assess their associations with various clinical, biochemical, and immunological parameters. We conducted comparative analyses to evaluate potential differences in clinical features and outcomes between SAOS-Ab-positive and SAOS-Ab-negative AE patient groups. These findings provide new insights into AE pathogenesis and may facilitate the identification of clinically distinct subgroups for personalized prognostic stratification.

This study aims to comprehensively determine the prevalence and clinical significance of SAOS-Ab in a large, multicenter cohort of AE patients. We seek to compare the demographic, clinical, paraclinical, and outcome features between SAOS-Ab-positive and SAOS-Ab-negative patients. Our findings aim to elucidate the role of systemic autoimmunity in AE pathogenesis and potentially identify a distinct patient subgroup requiring more aggressive initial therapy and vigilant long-term monitoring.

## Subjects and methods

### Study design and patient selection

This retrospective, multicenter cohort study enrolled consecutive patients newly diagnosed with AE between January 2018 and October 2022 at four tertiary medical centers in China: the First Affiliated Hospital of Henan University of Science and Technology, the People’s Hospital of Gaozhou, Beijing Tiantan Hospital, and Tianjin Medical University General Hospital. The study was approved by the Ethics Committee of The First Affiliated Hospital, and College of Clinical Medicine of Henan University of Science and Technology, and conducted in accordance with the ethical principles outlined in the Declaration of Helsinki, and all patients provided informed consent and approved participation (No. 2022-09-B067).

Inclusion criteria were: 1) over 18 years old; 2) meeting the diagnostic criteria for AE based on clinical signs and symptoms (15); 3) positive AE-related antibodies in serum and/or cerebrospinal fluid (CSF) tests; 4) complete baseline and clinical information; 5) informed consent for data collection and analysis. The exclusion criteria included: 1) pregnancy or lactation; 2) complicated with other acute neurological diseases; 3) with significant disability (modified Rankin scale[mRS] score ≥ 1) before; 4) active systemic infection, malignancy, or significant hematological disorders; 5) prior immunotherapy (corticosteroids, IVIG, plasmapheresis, rituximab, cyclophosphamide, etc.) within 4 weeks before admission.

### Data collection

A standardized data extraction form was developed to collect data from electronic medical records, including demographics, clinical manifestations, laboratory results (serum/CSF), mRS scores, clinical assessment scale in autoimmune encephalitis (CASE) scores, treatment, and prognosis. Certified neurologists conducted the diagnosis and treatment of all AE patients in accordance with the Chinese Expert Consensus on the Diagnosis and Management of Autoimmune Encephalitis (15).

Autoantibodies associated with AE were detected in both serum and CSF samples using a cell-based assay (CBA) with fixed transfected cells. All assays were performed at the KingMed Center for Clinical Laboratory (Guangzhou, China). Semi-quantitative titers were categorized as negative (<1:10), weakly positive (1:10-1:32), moderately positive (1:100-1:320), or strongly positive (≥1:1000) (16). ANA were detected by indirect immunofluorescence on HEp-2 cells (Euroimmun AG, Lübeck, Germany), with a positive cutoff set at >1:80. Anti-double-stranded DNA (anti-dsDNA) antibodies were also determined using indirect immunofluorescence (Euroimmun AG, Lübeck, Germany), with a positive cutoff set at >1:10. ENA, including anti-Smith antibody (Sm), Anti-Sjogren’s-Syndrome-related antigen A (SSA), Anti-Sjogren’s-Syndrome-related antigen B (SSB), Anti-Ro52 antibodies (Ro52), anti-ribonucleoprotein antibody (RNP), anti-histidyl-tRNA synthetase antibody (Jo-1), and anti-scleroderma 70 antibody (Scl-70), were detected by an immunoblotting assay (Euroimmun AG, Lübeck, Germany). The same platform was used to detect anti-mitochondrial antibody (AMA), Anti-polymyositis and scleroderma antibodies (PM-Scl), anti-histone, and anti-CENP-B antibodies. Anti-thyroglobulin (TG-Ab) and anti-thyroid peroxidase (TPO-Ab) antibodies were quantified using chemiluminescent immunoassays (Beckman Coulter, USA), with positive thresholds defined as > 12 IU/mL and > 60 IU/mL, respectively. Anticardiolipin antibodies (aCL) of IgG and IgM isotypes were measured by enzyme-linked immunosorbent assay (ELISA) (Euroimmun AG, Lübeck, Germany). Anti-neutrophil cytoplasmic antibodies (ANCA) were initially screened using indirect immunofluorescence, and specific reactivities to myeloperoxidase (MPO) and proteinase 3 (PR3) were subsequently confirmed with ELISA kits from Euroimmun AG. All laboratory procedures were rigorously conducted in accordance with the manufacturers’ standardized protocols. SAOS-Ab positivity was defined as the presence of at least one of the above autoantibodies meeting its respective clinical significance threshold. Based on this definition, patients were stratified into SAOS-Ab-positive and SAOS-Ab-negative groups.

The mRS and the CASE were pre-specified as primary clinical outcome measures owing to their established relevance in evaluating disease severity and predicting patient outcomes. The CASE score was used to assess the disease severity on admission. The total score of the CASE is 27 points. It consists of nine items: seizures, memory dysfunction, psychiatric symptoms, consciousness impairment, language problems, dyskinesia, gait instability and ataxia, brainstem dysfunction, and weakness (17). The mRS, which ranges from 0 (asymptomatic) to 6 (death), was employed to assess functional disability and to evaluate the outcome at discharge. Patient outcomes were assessed at hospital discharge using the mRS, with a score of ≥ 3 defined as a poor outcome and a score of < 3 as a good outcome (18). Admission to the intensive care unit (ICU) was deemed necessary if patients exhibited one or more of the following conditions: status epilepticus, delirium, coma, respiratory failure, severe sepsis, single or multiple organ failure, requirement for mechanical ventilation or vasopressor support, elevated APACHE II score, or other critical complications such as post-resuscitation status or increased intracranial pressure. The treatment for the enrolled patients encompassed four principal intervention strategies: anti-inflammatory (corticosteroids), IVIG, B cell targeted therapy (rituximab), plasmapheresis, and broad-spectrum immunosuppressants (including cyclophosphamide, cyclosporine, tacrolimus, and mycophenolate mofetil). All patients were followed up via telephone or interview for 24 months by independently blinded neurologists. A relapse was defined as the onset of new or recurrent neurological symptoms consistent with AE, not attributable to other causes, which led to a medical consultation resulting in a formal diagnosis in an outpatient or inpatient setting.

### Statistical analysis

Continuous variables’ differences between groups were analyzed using the Mann-Whitney U test for nonparametric parameters. Pearson’s chi-square test and Fisher’s exact test were employed to examine the differences in categorical variables’ proportions between groups. The Wilcoxon Rank Sum Test was utilized to investigate ranked data. Candidate predictors of recurrence were first evaluated using univariate Cox regression analysis, and factors significant in the univariate analysis were entered into a multivariable Cox regression model. The forward LR (forward stepwise regression based on maximum likelihood estimate) method was used to perform multivariable Cox regression analysis. Statistical analyses were performed using SPSS Statistics for Windows version 22, and figures were generated using GraphPad Prism 9.4. *p*-values less than 0.05 were considered indicative of statistical significance.

## Results

### Cohort characteristics and prevalence of SAOS-Ab in AE patients

**Figure 1:**
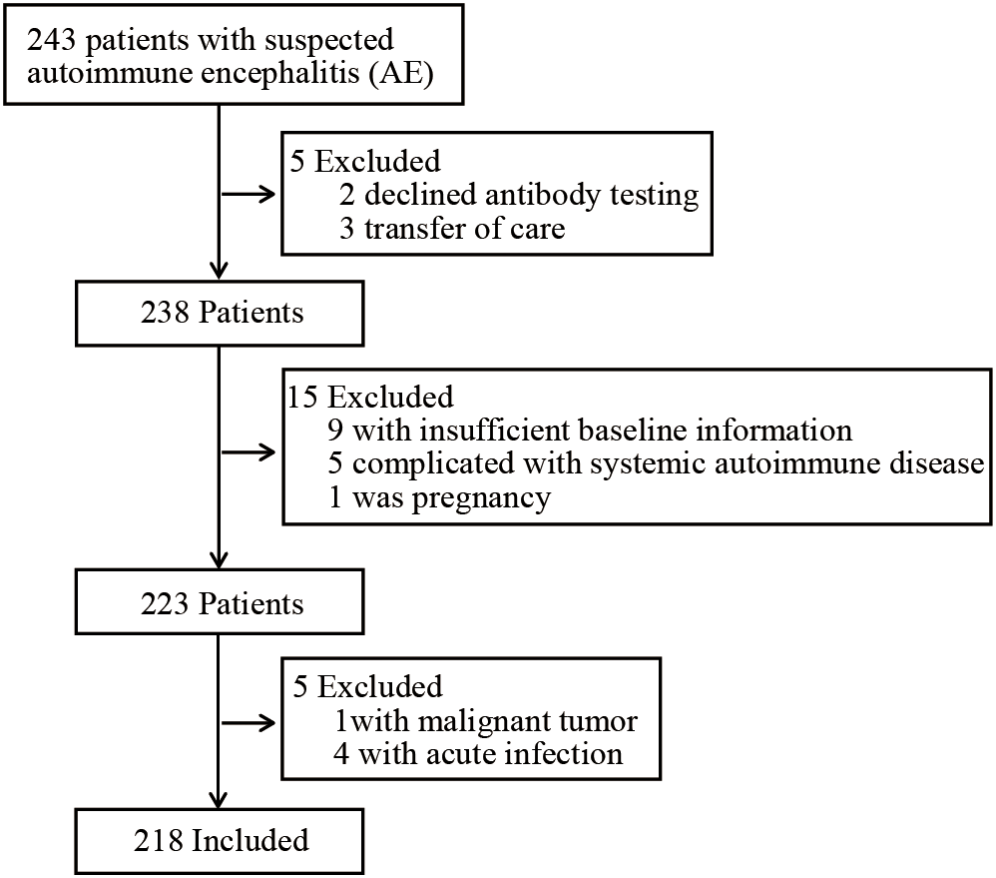
Flow chart of enrolled patients.

A total of 218 patients were enrolled in the study and 25 were excluded (**Figure 1**). The prevalence of SAOS-Ab seropositivity was 57.3% (125/218). The demographic and basic clinical characteristics of the two groups are summarized in **Table 1**. There was no significant difference in the median age at onset between the SAOS-Ab-positive (39, IQR: 21-56) and negative (40, IQR: 24-57) groups (*p* = 0.578). However, a significant gender disparity was observed (*p* = 0.004), with a higher proportion of females in the SAOS-Ab-positive group (55.2% vs. 35.5%). The distribution of specific neuronal autoantibody subtypes (e.g., anti-NMDAR, anti-LGI1) did not differ significantly between the two groups (*p* > 0.05 for all) (**Table 2**), indicating that the SAOS-Ab phenomenon is not restricted to a specific AE subtype. The spectrum of detected SAOS-Ab is detailed in **Table 1**. Among the 125 positive patients, the most frequently detected autoantibodies were TPO-Ab (56.0%), ANA (47.2%), TG-Ab (46.4%), and Ro52 (43.2%). A substantial proportion of patients (77.6%) had non-organ-specific antibodies, while 66.4% had organ-specific antibodies, with a significant overlap between the two groups. No significant differences were observed in the prevalence of comorbidities between the two groups, including coronary heart disease (CHD), hypertension, diabetes, hyperlipidemia, and chronic obstructive pulmonary disease (COPD) (**Table 1**).

**Table 1:** Demographics, paraclinical features, disease course and outcomes of AE patients.

Variables	SAOS-Ab-positive (N = 125)	SAOS-Ab-negative (N = 93)	*P* value
Demographics			
Age of onset, mean (IQR), y	39 (21–56)	40 (24-57)	0.578^a^
Sex, No. (%)			**0.004^b^**
Male	56 (44.8%)	60 (64.5%)	
Female	69 (55.2%)	33 (35.5%)	
Spectrum of SAOS-Ab	125	0	
ANA	59/125 (47.2%)	0	
dsDNA	1/125 (0.8%)	0	
ENA	67/125 (53.6%)	0	
Sm	2/125 (1.6%)	0	
SSA	37/125 (29.6%)	0	
SSB	4/125 (3.2%)	0	
Ro52	54/125 (43.2%)	0	
RNP	2/125 (1.6%)	0	
Jo-1	4/125 (3.2%)	0	
Scl-70	4/125 (3.2%)	0	
AMA	38/125 (30.4%)	0	
PM-SCL	5/125 (4.0%)	0	
Histone	3/125 (2.4%)	0	
CENP-B	5/125 (4.0%)	0	
Antithyroid antibodies	80/125 (64.0%)	0	
TG-Ab	58/125 (46.4%)	0	
TPO-Ab	70/125 (56.0%)	0	
Anticardiolipin antibodies	4/125 (3.2%)	0	
ANCA	4/125 (3.2%)	0	
Comorbidities ^d^			
Coronary heart disease	2/109 (1.8%)	4/83 (4.8%)	0.406^c^
Hypertension	20/109 (18.3%)	10/83 (12.0%)	0.234^b^
Diabetes	10/109 (9.20%)	10/83 (12.0%)	0.518^b^
Hyperlipidemia	7/109 (6.4%)	4/83 (4.8%)	0.760^c^
COPD	0/109 (0.0%)	0/83 (0.0%)	–
Clinical data at the first event			
CASE scores			**<0.0001^b^**
Mild:0-5	27/125 (21.6%)	53/93 (57.0%)	
Moderate:6-15	68/125 (54.4%)	32/93 (34.4%)	
Severe:16-27	30/125 (24.0%)	8/93 (8.6%)	
Admission to the ICU	27/125 (21.6%)	7/93 (7.5%)	**0.005^c^**
Auxiliary examination			
Laboratory Examinations at acute phase, mean (IQR)			
CD19^+^Bcells proportion, %^e^	17.66 (12.14-21.59)	13.79 (9.24-16.42)	**0.0027^a^**
CD3^+^Tcells proportion, %^f^	78.42 (73.36-85.25)	77.81 (73.70-84.36)	0.836^a^
CD4^+^Tcells proportion, %^f^	46.89 (37.28-54.24)	41.50 (36.57-46.78)	0.161^a^
CD8^+^Tcells proportion, %^f^	28.48 (22.60-38.22)	31.64 (26.50-39.65)	0.134^a^
Serum IgG, g/L^g^	14.96 (10.03-20.9)	8.11 (6.22-11.38)	**<0.0001^a^**
CSF IgG, g/L^h^	0.048 (0.023-0.051)	0.036 (0.0175-0.049)	0.1859^a^
Serum IgM, g/L^i^	1.25 (0.69-1.4)	0.87 (0.58-1.04)	**0.0334^a^**
Serum IgA, g/L^i^	2.20(1.47-2.76)	2.39 (1.61-2.81)	0.9935^a^
Serum IgE, IU/mL^j^	122.62(21.70-148)	126.19(17.20-156.46)	0.3747^a^
Inflammatory factors in serum, mean (IQR)			
TNF-α, pg/ml	8.07 (5.86-8.32)	7.94 (5.83-8.19)	0.909^a^
IL-6, pg/ml	5.17 (1.55-5.61)	5.21 (1.62-5.43)	0.889^a^
IL-8, pg/ml	23.37(9.51-33.6)	21.08 (7.88-30.35)	0.494^a^
IL-1β, pg/ml	3.08 (0-5.36)	2.59 (0-5.44)	0.62^a^
CRP	4.820 (0.305-9.57)	4.317 (0.153-10.17)	0.903^a^
Abnormal MRI	87/125 (69.60%)	60/93 (64.5%)	0.428^b^
Abnormal EEG	79/98 (80.6%)	52/63 (82.5%)	0.759^b^
Abnormal PET-CT	31/31 (100%)	29/30 (96.7%)	0.492^c^
Immune therapy at the first event			
Corticosteroids	112/125 (89.6%)	67/93 (72.0%)	**0.002^b^**
IVIG	96/125 (76.8%)	67/93 (72.0%)	0.618^a^
B cell targeted therapy	22/125 (17.6%)	5/93 (5.3%)	**0.006^b^**
plasmapheresis	2/125 (1.6%)	3/93 (3.2%)	0.654^c^
Immunosuppressant therapy	12/125 (9.6%)	6/93 (6.5%)	0.534^b^
**mRs at discharge**			**0.002^b^**
Good outcome: 0-2	66/125 (52.8%)	68/93 (73.1%)	
Poor outcome: 3-6	59/125 (47.2%)	25/93 (26.9%)	
Relapses over 24 months			
Number of patients who relapsed	36/121 (29.8%)	12/91 (13.2%)	**0.0043^b^**

AE, Autoimmune encephalitis; SAOS-Ab, Systemic and Organ-Specific antibodies; ANA, Antinuclear antibodies; dsDNA, double-stranded DNA; ENA, Anti-extractable nuclear antigen; Sm, anti-Smith antibody; SSA, Anti-Sjogren’s-Syndrome-related antigen A; SSB, Anti-Sjogren’s-Syndrome-related antigen B; Ro52, Anti-Ro52 antibody; RNP, anti-ribonucleoprotein antibody; Jo-1, anti-histidyl-tRNA synthetase antibody; Scl70, anti-scleroderma 70 antibody; AMA, Anti-mitochondrial antibody; PM-SCL, Anti-polymyositis and scleroderma antibody; CENP-B, Anti-centromere Protein B antibody; TG-Ab, Anti-thyroglobulin antibody; TPO-Ab, Anti-thyroid peroxidase antibody; ANCA, Antineutrophil cytoplasmic antibody; COPD, Chronic Obstructive Pulmonary Disease; TNF-α, Tumor necrosis factor-alpha, IL-6, Interleukin-6, IL-8, Interleukin-8, IL-1β, Interleukin-1beta; CRP, C Reactive Protein. CASE, Clinical Assessment Scale in Autoimmune Encephalitis; ICU, Intensive care unit; mRS, modified Rankin scale; IVIG, intravenous immunoglobulin. Bold text: *p* < 0.05; ^a^Mann-Whitney U-test; ^b^Pearson’s chi-square test; ^c^Fisher’s exact test for count data; A number of patients were not tested, and the actual sample sizes are: ^d^(SAOS-Ab-positive = 109, negative = 83); ^e^(SAOS-Ab-positive = 80, negative = 51); ^f^(SAOS-Ab-positive = 59, negative = 40); ^g^(SAOS-Ab-positive = 112, negative = 75); ^h^(SAOS-Ab-positive = 79, negative = 62); ^i^(SAOS-Ab-positive = 88, negative = 48); ^j^(SAOS-Ab-positive = 59, negative = 31).

**Table 2:** Subtypes and the antibody titers in AE patients.

Variables	SAOS-Ab-positive (N = 125)	SAOS-Ab-negative (N = 93)	*P* value
Subtypes of AE			
NMDAR	70 (56.0%)	51 (54.8%)	0.8645^a^
LGI1	33 (26.4%)	21 (22.6%)	0.5182^a^
GABA	5 (4.0%)	4 (4.3%)	> 0.99^b^
CASPR2	5 (4.0%)	4 (4.3%)	> 0.99^b^
AMPAR	2 (1.6%)	1 (1.1%)	> 0.99^b^
GAD65	6 (4.8%)	2 (2.2%)	0.428^b^
DPPX	0 (0.0%)	2 (2.2%)	0.1809^b^
mGluR1	1 (0.8%)	1 (1.1%)	> 0.99^b^
mGluR5	0 (0.0%)	1 (1.1%)	0.4266^b^
IgLON5	0 (0.0%)	2 (2.2%)	0.1809^b^
GlyR1	1 (0.8%)	0 (0.0%)	> 0.99^b^
Amphiphysin	1 (0.8%)	2 (2.2%)	0.5768^b^
CV2	1 (0.8%)	1 (1.1%)	> 0.99^b^
Yo	0 (0.0%)	1 (1.1%)	0.4266^b^
Serum autoantibody titers of AE			0.870^a^
Negative	40/125 (32.0%)	30/93 (32.3%)	
Weakly positive (1:10-1:32)	56/125 (44.8%)	39/93 (41.9%)	
Moderately positive (1:100-1:320)	26/125 (20.8%)	20/93 (21.5%)	
Strongly positive (≥1:1000)	3/125 (2.4%)	4/93 (4.3%)	
CSF autoantibody titers of AE			0.034^a^
Negative	12/125 (9.6%)	16/93 (17.2%)	
Weakly positive (1:1-1:3.2)	30/125 (24.0%)	27/93 (29.0%)	
Moderately positive (1:10-1:32)	39/125 (31.2%)	33/93 (35.5%)	
Strongly positive (≥1:100)	44/125 (35.2%)	17/93 (18.3%)	

AE, Autoimmune encephalitis; SAOS-Ab, Systemic and organ-specific antibodies; NMDAR, N-methyl-D-aspartate receptor; LGI1, Leucine-rich glioma-inactivated 1; GABA; γ-Aminobutyric acid receptor; CASPR2, Contactin-associated protein-like 2; AMPAR, α-Amino-3-hydroxy-5-methyl-4-isoxazolepropionic acid receptor; GAD65, Glutamic acid decarboxylase 65; DPPX, Dipeptidyl-peptidase-like protein 6; mGluR1, Metabotropic glutamate receptor 1; mGluR5, Metabotropic glutamate receptor 5; IgLON5, IgLON family member 5; GlyR1, Glycine receptor α1 subunit; CV2, Contactin-2; Yo, Cerebellar degeneration-related protein 2. Bold text, *p* < 0.05; ^a^Pearson’s chi-square test, ^b^Fisher’s exact test for count data.

### SAOS-Ab-positive AE patients exhibited a distinct immune profile

**Figure 2:**
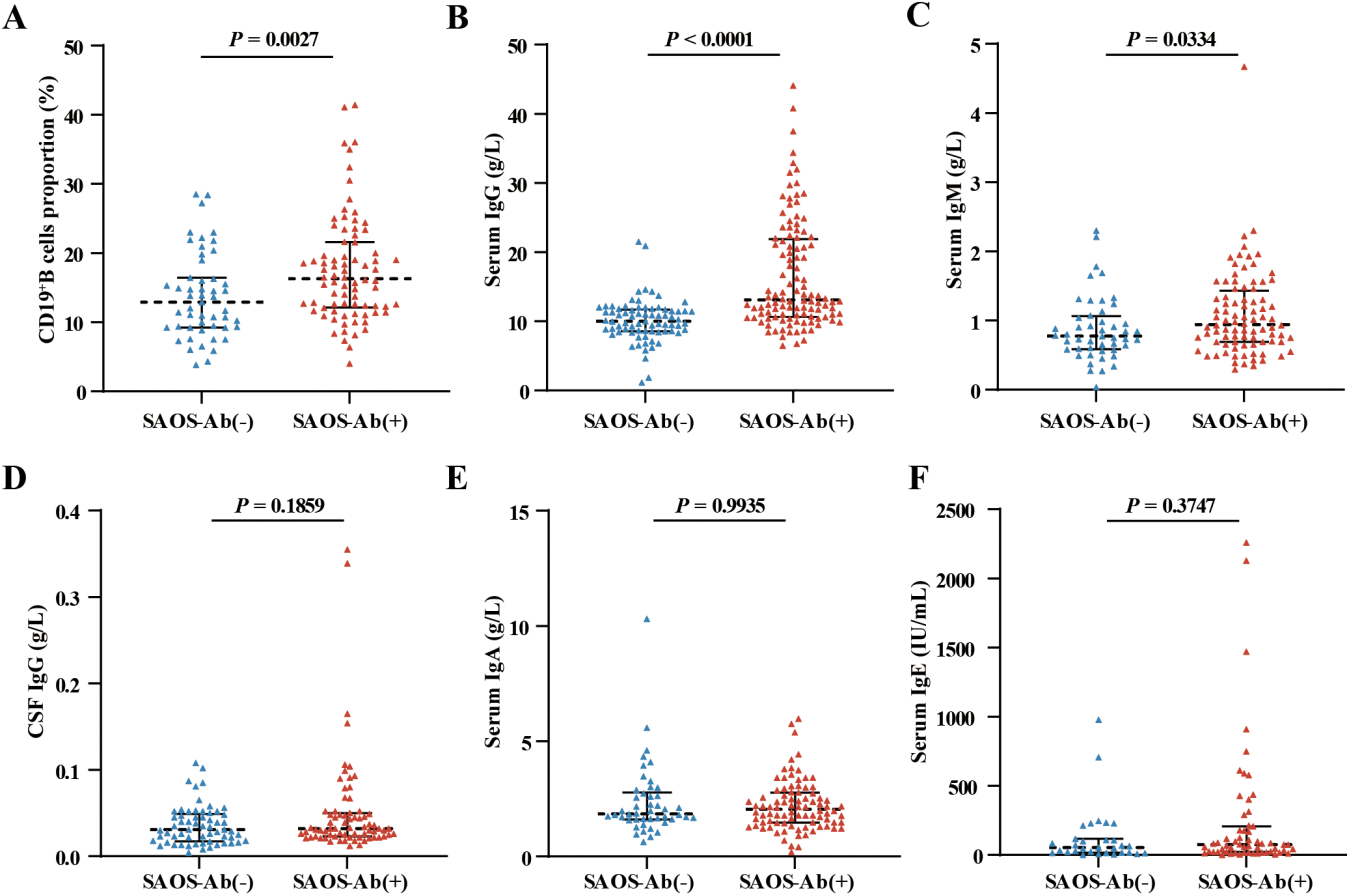
Paraclinical immunological profiles between SAOS-Ab-positive and SAOS-Ab-negative AE patients. **(A–C)** The SAOS-Ab-positive group exhibited significantly higher levels of serum CD19+ B cells, serum IgG, and serum IgM compared to the SAOS-Ab-negative group (*p* < 0.05 for all). **(D–F)** In contrast, no statistically significant differences were observed between the two groups in CSF IgG, serum IgA, and serum IgE levels (*p* > 0.05 for all). Data are presented as median with interquartile range (IQR) and analyzed using the Mann-Whitney U test.

Comprehensive immunological profiling revealed significant differences between the groups. Patients in the SAOS-Ab-positive group exhibited markedly higher levels of the proportion of CD19^+^ B lymphocytes (17.66% vs. 13.79%, *p* = 0.0027), IgG (median 14.96 g/L vs. 8.11 g/L, *p* < 0.0001) and IgM (median 1.25 g/L vs. 0.87 g/L, *p* = 0.0334) in peripheral blood (**Figures 2A–C**). In contrast, no significant differences were observed in CSF IgG, serum IgA and serum IgE levels (*p* > 0.05 for all) (**Figures 2D–F**). Levels of systemic inflammatory markers, including tumor necrosis factor-alpha (TNF-α), interleukin-6 (IL-6), interleukin-8 (IL-8), interleukin-1beta (IL-1β), and C-reactive protein (CRP), were also comparable between groups (**Table 1**). Notably, while serum titers of the primary neuronal autoantibodies were similar, CSF titers were significantly higher in the SAOS-Ab-positive cohort (*p* = 0.034) (**Table 2**), suggesting a possible link between systemic immune activation and intrathecal antibody production.

### SAOS-Ab-positive AE patients exhibited more severe clinical manifestations and worse clinical outcomes

**Figure 3:**
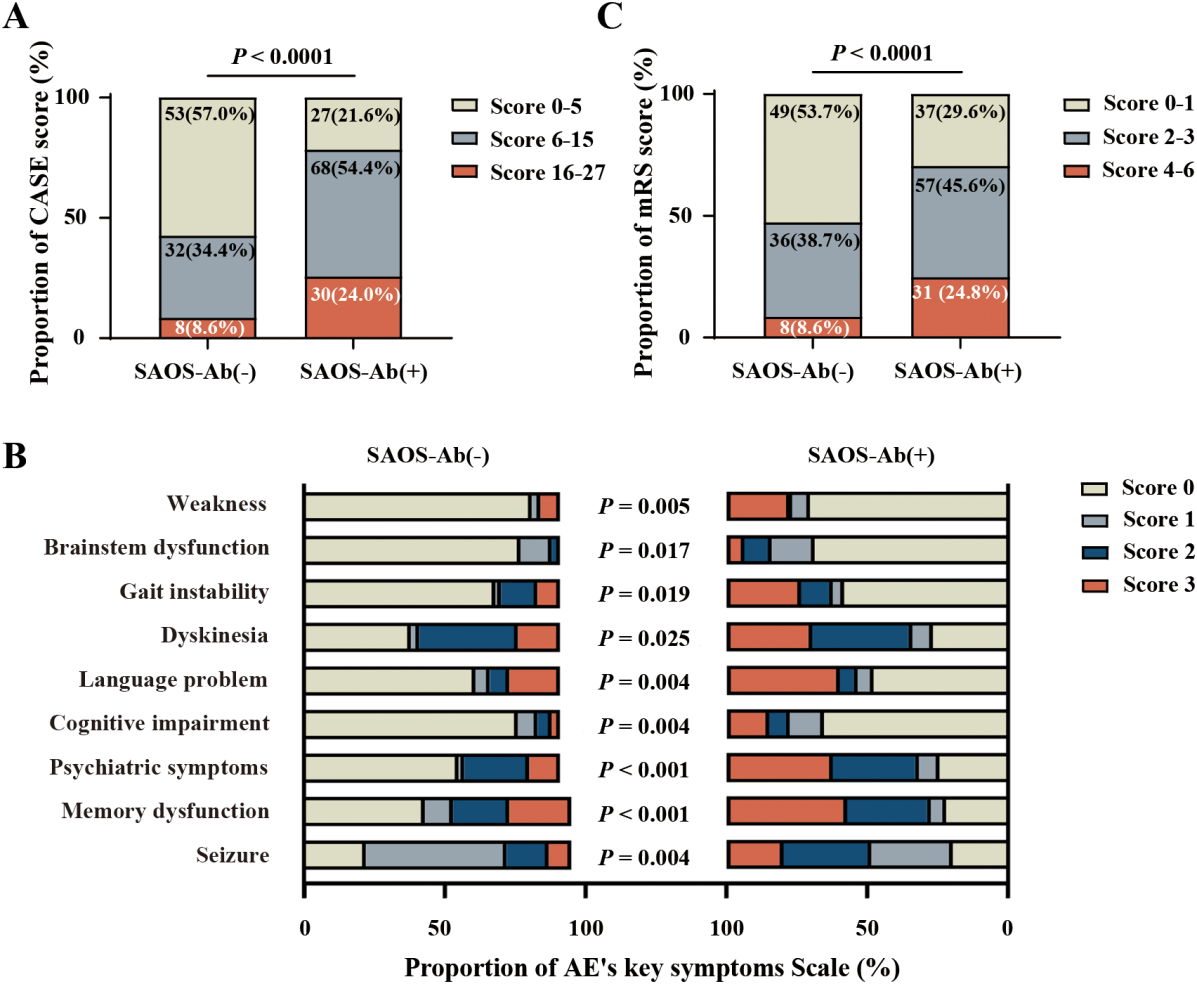
Comparison of the clinical severity and outcomes between SAOS-Ab-positive and SAOS-Ab-negative AE patients. **(A)** The CASE score distribution on admission was significantly different between the two groups, and the SAOS-Ab-positive cohort showed a markedly higher score (*p* < 0.0001). **(B)** The CASE score revealed that patients in the SAOS-Ab-positive group had significantly higher severity scores across the key clinical symptoms compared to the SAOS-Ab-negative group (*p* < 0.05 for all). **(C)** The distribution of mRS scores at discharge was significantly different between groups, and the SAOS-Ab-positive group showed a markedly higher score (*p* < 0.0001).

Disease severity at onset was significantly greater in SAOS-Ab-positive patients, as reflected by higher CASE scores (*p* < 0.0001) (**Figure 3A**). A greater proportion of these patients were classified as having severe disease (24.0% vs. 8.6%), and a smaller proportion had mild disease (21.6% vs. 57.0%) compared to the SAOS-Ab-negative group (**Figure 3A**). This increased severity was reflected in the clinical symptoms. SAOS-Ab-positive patients had a significantly higher CASE scores in weakness (28.0% vs 10.8%; *p* = 0.005), brainstem dysfunction (47.2% vs 20.4%; *p* = 0.017), gait instability and ataxia (40.0% vs 24.7%; *p* = 0.019), dyskinesia (71.2% vs 57.0%; *p* = 0.025), language problems (50.4% vs 32.3%; *p* = 0.004), cognitive impairment (32.9% vs 16.1%; *p* = 0.004), psychiatric symptoms (73.6% vs 38.7%; *p* < 0.001), memory dysfunction (76.8% vs 25.6%; *p* < 0.001), seizures (79.2% vs 35.5%; *p* = 0.004) (**Figure 3B**). Furthermore, a significantly higher rate of ICU admission was observed among SAOS-Ab-positive patients at the time of hospital admission(21.6% vs 7.5%; *p* = 0.005) (**Table 1**).

**Figure 4:**
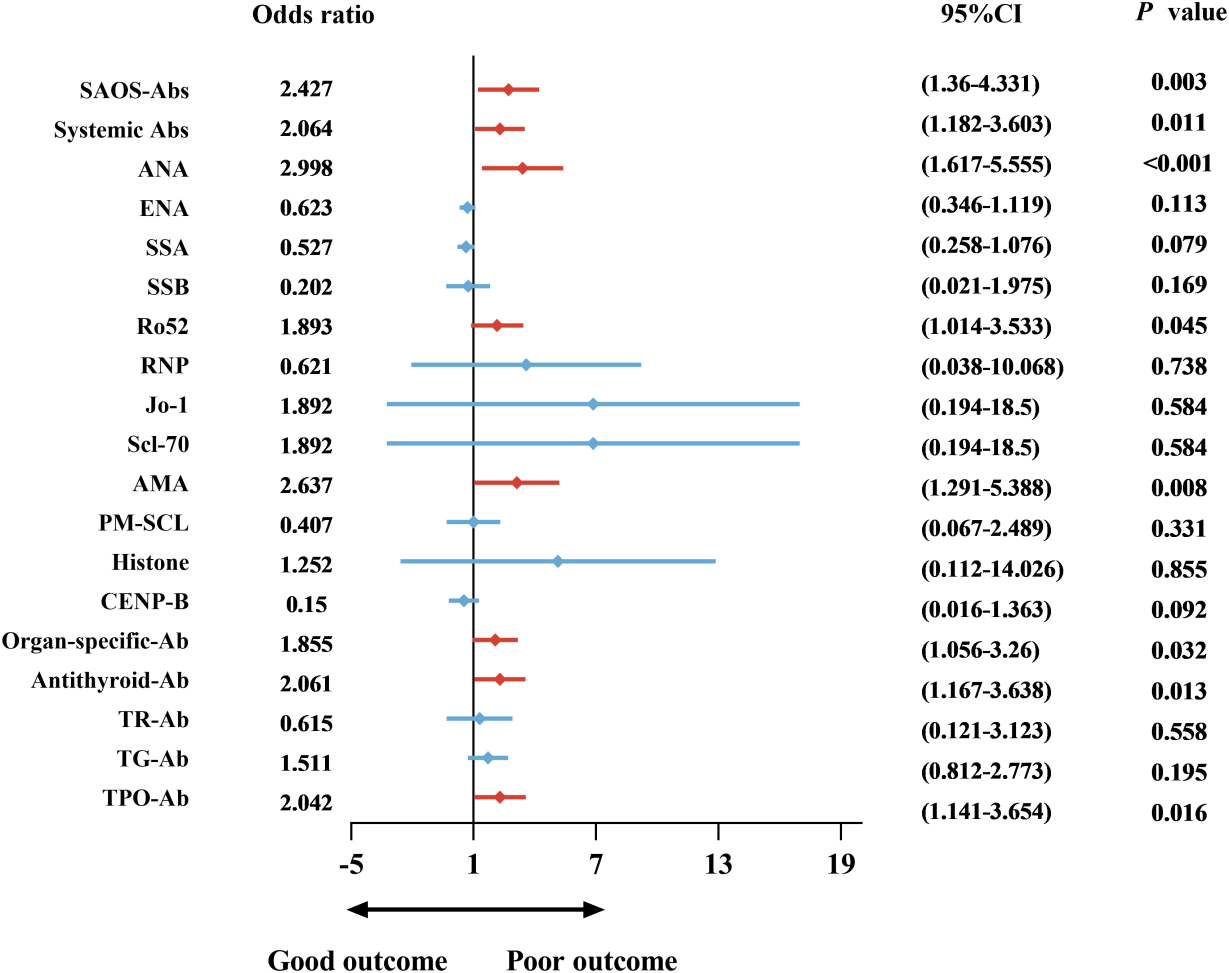
Forest plot of the association between SAOS-Ab and poor functional outcome in AE patients. The plot displays the results of a multivariable logistic regression analysis, presenting adjusted OR with corresponding 95% CI for the association between various SAOS-Ab and poor outcome, defined as a mRS score ≥ 3 at discharge.

Treatment patterns differed between the groups. SAOS-Ab-positive patients received first-line corticosteroids (89.6% vs. 72.0%, *p* = 0.002) and second-line B cell targeted therapy (17.6% vs. 5.3%, *p* = 0.006) more frequently (**Table 1**). At discharge, the functional outcome was significantly worse in SAOS-Ab-positive patients, as reflected by higher mRS scores (*p* < 0.0001) (**Figure 3C**). A poor outcome (mRS 3-6) was observed in 47.2% (59/125) of SAOS-Ab-positive patients compared to 26.9% (25/93) of SAOS-Ab-negative patients (*p* = 0.002) (**Table 1**). Univariable binary logistic regression analysis identified a significant association between SAOS-Ab positivity and poor outcome (OR: 2.427, 95% CI: 1.36-4.331, *p* = 0.003), with specific antibodies including ANA (OR: 2.998, 95% CI: 1.617-5.555, *p* < 0.001), Ro52 (OR: 1.893, 95% CI: 1.014-3.533, *p* = 0.045), and AMA (OR: 2.637, 95% CI: 1.291-5.388, *p* = 0.008) also demonstrating significant associations. Among organ-specific autoantibodies, both antithyroid antibodies (OR: 2.061, 95% CI: 1.167-3.638, *p* = 0.013) and TPO antibodies (OR: 2.042, 95% CI: 1.141-3.654, *p* = 0.016) were significantly associated with poor outcomes (**Figure 4**).

### Positivity of SAOS-Ab is an independent risk factor for relapse in AE

Kaplan-Meier analysis for relapse risk was performed in AE patients starting from the time of discharge. During a median follow-up of 18.2 months (IQR: 5.7-24.0), 4 patients (3.20%) were lost to follow-up in the SAOS-Ab-positive group, compared to 2 patients (2.15%) in the SAOS-Ab-negative group (**Table 1**). This attrition rate of below 10% in both cohorts is unlikely to have introduced significant bias into the comparative analysis. Among those patients, 48 had experienced relapse, while 164 had achieved remission (77.4%). Compared to the SAOS-Ab-negative group, the relapse rate was significantly higher in the SAOS-Ab-positive group (29.8% vs. 13.2%, *p* = 0.0043) (**Table 1**).

**Figure 5:**
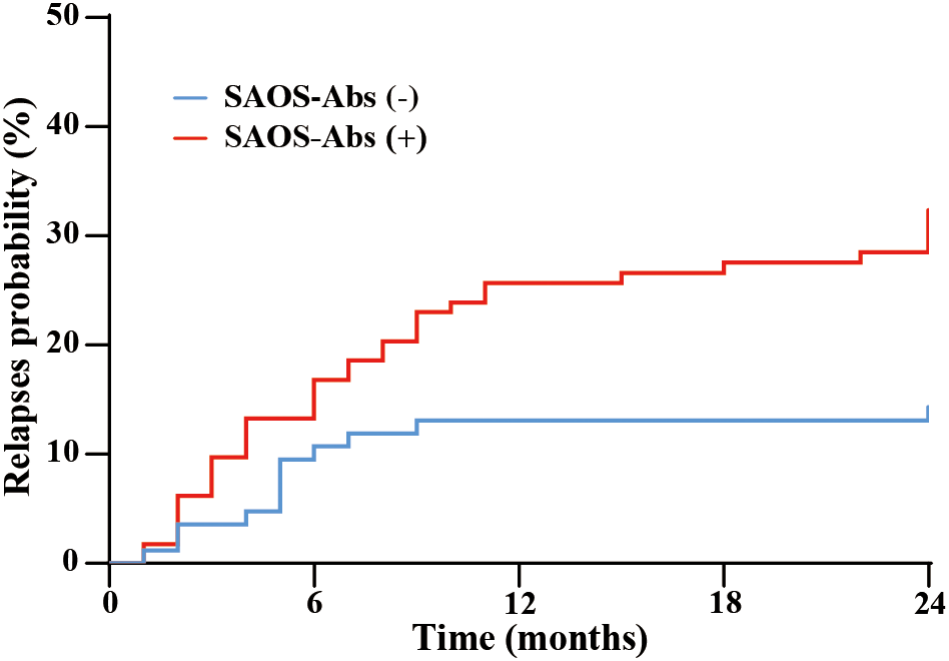
The comparison of the relapse probability between SAOS-Ab-positive and SAOS-Ab-negative AE patients. Kaplan-Meier analysis demonstrated a significantly higher relapse rate in the cohort with SAOS-Ab positivity over 24 months of follow-up.

Candidate predictors of recurrence were initially evaluated using univariate Cox proportional hazards regression analysis, including age at onset, gender, CASE scores, mRS scores, ICU admission, and treatment regimens. A significantly elevated relapse risk was observed in the SAOS-Ab-positive group (HR: 2.439, 95% CI: 1.269-4.690, *p* = 0.008) and among patients who did not receive immunosuppressant therapy (HR: 0.336, 95% CI: 0.157-0.7195, *p* = 0.005). In contrast, no significant associations were found for corticosteroids (*p* = 0.057), IVIG (*p* = 0.302), B cell targeted therapy (*p* = 0.748), or plasmapheresis (*p* = 0.642). Variables significant in the univariate analysis were entered into a multivariable Cox regression model (forward likelihood ratio method). In the multivariable Cox regression model, after adjusting for established risk factors, positivity for SAOS-Ab remained an independent risk factor for relapse (adjusted HR: 2.270, 95% CI: 1.174-4.387, *p* = 0.015) (**Figure 5**).

## Discussion

This multicenter retrospective cohort study delineates the substantial clinical and immunological implications of SAOS-Ab coexistence in AE. We demonstrated that SAOS-Ab, detectable in over half of the AE patients, is associated with enhanced systemic immune activation, greater neurological severity, poorer functional outcomes, and a significantly elevated relapse risk. These findings expand our understanding of AE immunopathogenesis and offer actionable clinical insights for effective risk stratification and clinical management.

Multiple autoimmune syndromes (MAS) and overlap syndromes (OS) are the most frequently observed associations between autoimmune diseases (19). Patients diagnosed with a single autoimmune disease often exhibit specific autoantibodies associated with other autoimmune conditions, which is a common phenomenon (20, 21). While clinical evidence, shared pathogenic mechanisms, and pleiotropic effects of non-disease-specific genes suggest a common etiological background for autoimmune conditions, the patterns of autoimmune comorbidity remain incompletely elucidated (22, 23). Our study reveals a high prevalence of SAOS-Ab positivity, a figure that exceeds prior reports (13, 14). Prior studies have predominantly centered on patients with concurrent overt systemic autoimmune diseases, rather than serological autoimmunity in the absence of clinical manifestations. Our study employed a comprehensive screening panel encompassing both organ-specific and non-organ-specific autoantibodies. Indeed, autoantibodies specific to many autoimmune diseases may be detectable in serum years before the onset of typical clinical symptoms. The female predominance (55.2%) among SAOS-Ab-positive patients aligns with the established female preponderance in systemic autoimmune diseases, potentially implicating estrogen-mediated immunomodulation (24). Significantly, SAOS-Ab positivity was not restricted to any specific neuronal autoantibody subtype, indicating it is a cross-cutting phenomenon in AE immunopathology.

Immunologically, SAOS-Ab-positive patients exhibited elevated serum IgG and IgM levels, as well as an increased proportion of B cells, indicating a heightened systemic humoral immune response. The development of autoreactive T and B cells, along with the production of polyclonal autoantibodies, constitutes a fundamental mechanism in the pathogenesis of autoimmune disorders (25). B cells contribute to AE through multiple mechanisms, including the secretion of neuronal autoantibodies, the production of proinflammatory cytokines, and antigen presentation to T cells (26). A previous study demonstrated that B cell activation promotes the accumulation of intrathecal plasmablasts and antibody production in anti-NMDAR encephalitis (27). This seems to explain the finding of higher CSF titers of neuronal autoantibodies in the SAOS-Ab-positive group.

Clinically, SAOS-Ab-positive AE patients presented with greater neurological severity, including higher mRS and CASE scores, a higher frequency of seizures, psychiatric symptoms, cognitive impairment, and increased ICU admission rates. This aligns with reports in anti-NMDAR encephalitis, where concomitant ANA positivity was linked to more severe manifestations and a higher incidence of epilepsy, an association sometimes attributed to more profound blood-brain barrier (BBB) disruption (13, 14). Previous studies have identified several predictors of relapse in AE, including older age, sex, the presence of status epilepticus, CSF abnormalities, the absence of second-line immunotherapy, MRI changes, and specific neuronal autoantibody subtypes (28–30). Our research further found that the SAOS-ab antibody is an independent risk factor for recurrence. The mechanisms underlying the more severe phenotype and higher relapse risk in SAOS-Ab-positive AE patients likely involve a synergistic interplay between systemic immune activation and CNS vulnerability. B cell dysregulation and polyclonal antibody production may not only amplify the intrathecal pathogen-specific immune response but also contribute to the injury of the BBB, leading to more severe nerve damage. Additionally, some SAOS-Ab may cross-react with CNS antigens, which could directly worsen neural injury. Future experimental studies are needed to clarify the underlying mechanisms.

Several limitations of this investigation warrant consideration. First, the retrospective study design may introduce potential selection and information biases. Second, the absence of a healthy control group restricts our ability to establish normative comparisons. Third, variations in treatment protocols across centers may affect data consistency. Furthermore, the reliance on telephone-based follow-up evaluations may compromise the precision of clinical outcome measurements. These methodological constraints highlight the need for future prospective studies incorporating standardized protocols to more rigorously evaluate the clinical significance and pathophysiological role of SAOS-Abs in AE. Future prospective studies are warranted to elucidate the precise mechanisms by which SAOS-Ab contribute to AE pathogenesis and to validate their utility as biomarkers for stratification of disease severity and individualized treatment strategies.

## Conclusion

In summary, our findings demonstrate that SAOS-Abs exhibit significant prevalence among patients with AE. SAOS-Ab-positive AE patients presented more severe symptoms, including higher CASE and mRS scores, and higher rates of ICU admission. Additionally, SAOS-Ab positivity was identified as an independent predictor of poorer functional recovery and a higher risk of relapse. These findings highlight SAOS-Ab as a potential prognostic biomarker in AE. Early identification of these patients may enable risk stratification, guiding therapeutic strategies and closer clinical follow-up to mitigate the risk of relapse.

## Data Availability

The original contributions presented in the study are included in the article/supplementary material. Further inquiries can be directed to the corresponding authors.
